# Protective Anti-Inflammatory and Antioxidant Mechanisms of *Ohwia caudata* Leaf Hydroethanolic Extract in a Dermatitis Mouse Model

**DOI:** 10.3390/life15111707

**Published:** 2025-11-04

**Authors:** Tzu-Kai Lin, Bruce Chi-Kang Tsai, Shih-Wen Kao, Chia-Lun Tsai, Chia-Hua Kuo, Tsung-Jung Ho, Dennis Jine-Yuan Hsieh, Shinn-Zong Lin, Wei-Wen Kuo, Chih-Yang Huang

**Affiliations:** 1 Department of Dermatology, Hualien Tzu Chi Hospital, Buddhist Tzu Chi Medical Foundation, Hualien 970473, Taiwan; 2 Cardiovascular and Mitochondrial Related Disease Research Center, Hualien Tzu Chi Hospital, Buddhist Tzu Chi Medical Foundation, Hualien 970473, Taiwan; 3 Department of Orthopedic Surgery, Chung Shan Medical University Hospital, Taichung 402306, Taiwan; 4 School of Medicine, Chung Shan Medical University, Taichung 402306, Taiwan; 5 Laboratory of Exercise Biochemistry, The Education University of Hong Kong, New Territories, Hong Kong; 6 School of Physical Education and Sports Science, Soochow University, Suzhou 215006, China; 7 Department of Movement Sciences and Sports Training, School of Sport Sciences, University of Jordan, Amman 11942, Jordan; 8 Department of Chinese Medicine, Hualien Tzu Chi Hospital, Buddhist Tzu Chi Medical Foundation, Hualien 970473, Taiwan; 9 Integration Center of Traditional Chinese and Modern Medicine, Hualien Tzu Chi Hospital, Buddhist Tzu Chi Medical Foundation, Hualien 970473, Taiwan; 10 Department of Medical Laboratory and Biotechnology, Chung Shan Medical University, Taichung 402306, Taiwan; 11 Clinical Laboratory, Chung Shan Medical University Hospital, Taichung 402306, Taiwan; 12 Bioinnovation Center, Buddhist Tzu Chi Medical Foundation, Hualien 970473, Taiwan; 13 Department of Neurosurgery, Hualien Tzu Chi Hospital, Buddhist Tzu Chi Medical Foundation, Hualien 970473, Taiwan; 14 Department of Biological Science and Technology, College of Life Sciences, China Medical University, Taichung 406040, Taiwan; 15 Ph.D. Program for Biotechnology Industry, China Medical University, Taichung 406040, Taiwan; 16 School of Pharmacy, China Medical University, Taichung 406040, Taiwan; 17 Graduate Institute of Biomedical Sciences, China Medical University, Taichung 404333, Taiwan; 18 Department of Medical Research, China Medical University Hospital, China Medical University, Taichung 404327, Taiwan; 19 Department of Medical Laboratory Science and Biotechnology, Asia University, Taichung 413305, Taiwan

**Keywords:** *Ohwia caudata*, dermatitis, inflammation, anti-oxidation, skin care

## Abstract

Contact dermatitis is a common inflammatory skin disorder characterized by erythematous and pruritic lesions caused by irritant exposure. *Ohwia caudata*, a traditional medicinal plant, possesses anti-inflammatory and antioxidant properties, but its dermatoprotective potential remains unclear. To investigate the protective effects and mechanisms of *Ohwia caudata* leaves’ hydroethanolic extract in a murine model of TPA-induced contact dermatitis. Major phytochemicals in *Ohwia caudata* extract were identified by HPLC–MS. SKH1/J hairless mice were topically treated with *Ohwia caudata* extract following TPA stimulation. Skin barrier function was assessed by transepidermal water loss and hydration. Inflammatory (IL-6, TNF-α, TGF-β), antioxidant (SIRT1, Nrf2, HO-1), and ERK-related (p-ERK, eIF2α, ATF-4, CHOP) proteins were analyzed by immunoblotting and immunofluorescence. HPLC–MS revealed harmine, swertisin, isoliquiritigenin, eupatilin, 3′,4′-dimethoxyflavone, and nerolidol as key constituents. The extract significantly reduced transepidermal water loss and enhanced hydration, indicating improved barrier integrity. It downregulated IL-6 and TNF-α while restoring TGF-β expression. ERK and downstream eIF2α/ATF-4/CHOP activation were suppressed, whereas SIRT1/Nrf2/HO-1 antioxidant signaling was enhanced. *Ohwia caudata* leaves hydroethanolic extract protects against TPA-induced dermatitis by improving skin barrier function, suppressing inflammation, and activating antioxidant defense, supporting its potential as a natural therapeutic for inflammatory skin diseases.

## 1. Introduction

The skin, which is composed of three primary layers—the epidermis, the dermis, and the hypodermis—represents the largest and one of the most complex organs in the human body. It functions as the first line of defense, forming an essential boundary between internal physiological environment in the body and the constantly changing external surroundings. It plays several key roles, such as providing a protective barrier, regulating homeostasis, and defending against various environmental stressors [1]. Contact dermatitis is a common inflammatory skin condition frequently observed in clinical settings. It manifests as itchy, red, and occasionally blistering lesions that develop after the skin comes into direct contact with irritating or allergenic chemical or physical substances. The disease involves a multifaceted biological response, including impairment of the skin’s barrier function, stimulation of both innate and adaptive immune systems, and subsequent production of proinflammatory cytokines and mediators that contribute to inflammation and tissue damage [2,3,4]. 12-O-tetradecanoylphorbol-13-acetate (TPA) is a widely used chemical compound that serves as a standard experimental agent for studying the molecular and cellular processes underlying skin inflammation and carcinogenesis [5,6]. In murine models, TPA has been widely used to induce acute dermatitis, inflammation, and edema during the tumor promotion stage, thereby providing a reliable system for evaluating therapeutic interventions targeting inflammatory skin disorders [7,8,9].

Traditional Chinese medicines and their bioactive components have long been recognized for their therapeutic potential in promoting health and preventing diseases. Traditional Chinese medicines help the body resist various pathological conditions and external stressors, supporting physiological balance and enhancing resilience against environmental or disease-related challenges [10,11,12,13,14,15]. A wide range of traditional medicinal plants exert beneficial effects on the skin, including wound-healing, moisturizing, antioxidant, anti-inflammatory, and anti-aging properties. Their natural origin, safety, and multifunctionality make them valuable resources for developing effective and sustainable pharmaceutical and cosmetic formulations. Continued research on their standardization, safety, and clinical efficacy is essential to fully realize their potential in modern skin treatment and care [16,17]. *Ohwia caudata* (syn. *Desmodium caudatum*), a member of the Fabaceae family, is a well-known traditional Chinese medicine utilized in the treatment of febrile illnesses, colds, icterohepatitis, gastroenteritis, bacillary dysentery, and rheumatic arthritis [18,19]. Phytochemical analyses have revealed that *Ohwia caudata* contains alkaloids, triterpenoids, and flavonoids, the latter exhibiting strong anti-inflammatory and antioxidative activities [20,21]. Moreover, *Ohwia caudata* has been reported to mitigate Alzheimer’s disease–related pathology [22], attenuate doxorubicin-induced cytotoxicity [23,24,25], and inhibit influenza A virus infection [19]. Given its diverse pharmacological properties, *Ohwia caudata* represents a promising candidate for developing novel therapeutic agents targeting inflammatory and oxidative stress–related skin diseases. However, its potential efficacy against contact dermatitis has not been fully elucidated.

Previous studies have identified several health benefits associated with *Ohwia caudata*. However, to the best of our knowledge, most previous studies have focused on the pharmacological activities and therapeutic properties of the water extract of *Ohwia caudata*. In contrast, little attention has been given to its leaf hydroethanolic extract. The hydroethanolic extract was selected because ethanol efficiently extracts both polar and non-polar bioactive compounds, which are less soluble in water. These compounds may exhibit enhanced antioxidant and anti-inflammatory activities, which are advantageous for skin protection. Therefore, the objective of this study was to investigate the protective potential of the hydroethanolic extract derived from *Ohwia caudata* leaves using a mouse model of contact dermatitis. The hypothesis was that treatment with this leaf hydroethanolic extract could alleviate TPA-induced skin injury by reducing inflammation and oxidative stress, thereby restoring skin integrity and overall barrier function. The results demonstrated that *Ohwia caudata* leaf hydroethanolic extract alleviated acute contact dermatitis through inhibition of inflammatory pathways and enhancement of antioxidant defense mechanisms, thereby highlighting its potential as a therapeutic candidate for inflammatory skin disorders.

## 2. Materials and Methods

### 2.1. Materials

Unless otherwise indicated, all analytical-grade chemicals used in this study were purchased from Sigma-Aldrich (St. Louis, MO, USA) and Merck (Darmstadt, Germany). Dried, healthy, and mature leaves of *Ohwia caudata* (Mo Cao; Family: Fabaceae; *Ohwia caudata* (Thunb.) H. Ohashi; taxonomic synonym: *Desmodium caudatum* (Thunb. ex Murray) DC.) were authenticated and obtained from the Department of Chinese Medicine, Hualien Tzu Chi Hospital (Hualien, Taiwan). TPA [purity ≥ 99.0% (TLC); cat. no. P8139] was acquired from Sigma-Aldrich.

Primary antibodies were obtained from various suppliers. Anti-phosphorylated extracellular signal-regulated kinase (p-ERK; cat. no. sc-7383), anti-transforming growth factor beta (TGF-β; cat. no. sc-130348), anti-tumor necrosis factor-alpha (TNF-α; cat. no. sc-52746), anti-Sirtuin 1 (SIRT1; cat. no. sc-74465), anti-interleukin-6 (IL-6; cat. no. sc-130326), anti-heme oxygenase-1 (HO-1; cat. no. sc-136961), and anti-β-actin (cat. no. sc-47778) were from Santa Cruz Biotechnology (Santa Cruz, CA, USA). Antibodies against phosphorylated eukaryotic initiation factor 2 (p-eIF2α; cat. no. #9721), C/EBP homologous protein (CHOP; cat. no. #2895), and activating transcription factor 4 (ATF-4; cat. no. #11815) were purchased from Cell Signaling Technology (Danvers, MA, USA). Anti-nuclear factor erythroid 2-related factor 2 (Nrf2; cat. no. ab89443) was obtained from Abcam (Cambridge, UK). Horseradish peroxidase–conjugated secondary antibodies were from Santa Cruz Biotechnology, and Alexa Fluor^®^ 488 goat anti-mouse IgG (A11001) and Alexa Fluor^®^ 488 goat anti-rabbit IgG (A11008) were purchased from Thermo Fisher Scientific (Waltham, MA, USA).

### 2.2. Preparation of Ohwia caudata Leaf Hydroethanolic Extract

A total of ten grams of dried *Ohwia caudata* leaves were washed, air-dried, and ground into a fine powder. The powder was extracted with 200 mL of 75% ethanol by sonication for 1 h, followed by filtration and centrifugation at 10,000 rpm for 15 min at 4 °C. The supernatant was collected and stored at 4 °C until use. The crude extract concentration was determined after vacuum drying. The phytochemical composition of the hydroethanolic extract was analyzed by high-performance liquid chromatography coupled with tandem mass spectrometry (HPLC–MS), with technical assistance from Protech Technology Enterprise Co., Ltd. (Taipei, Taiwan).

### 2.3. Animal Experiment Design

All animal experiments were approved by the Institutional Animal Care and Ethics Committee of Hualien Tzu Chi Hospital (Hualien, Taiwan; approval no. 111-37) and conducted in accordance with the NIH Guide for the Care and Use of Laboratory Animals. This study was performed in compliance with the ARRIVE guidelines (https://arriveguidelines.org, accessed on 1 December 2022). Female SKH1/J hairless mice (8 weeks old) were obtained from BioLASCO Taiwan Co., Ltd. (Taipei, Taiwan) and housed at the Laboratory Animal Center of Tzu Chi University under controlled conditions (22 ± 2 °C, 50 ± 5% humidity, 12 h light/dark cycle) with ad libitum access to standard chow (Lab Diet 5001; PMI Nutrition International Inc., Brentwood, MO, USA) and reverse osmosis water. After acclimatization, mice (*n* = 18) were randomly assigned to three groups (*n* = 6 each): control, TPA-stimulated, and TPA-stimulated plus *Ohwia caudata* leaf hydroethanolic extract treatment. Acute contact dermatitis was induced by daily topical application of 60 μL of 0.001% TPA to the dorsal skin. Three hours after TPA stimulation, 60 μL of *Ohwia caudata* leaf hydroethanolic extract (30 mg/mL) was applied twice daily for six hours to the treatment group, while the TPA group received vehicle only. Treatments were continued for seven days. On day eight, transepidermal water loss (TEWL), melanin index, and stratum corneum hydration were measured using a multi-probe adapter system (Courage + Khazaka, Cologne, Germany) prior to sacrifice. Dorsal skin samples were collected for further analysis after euthanasia.

### 2.4. Frozen Section

Back skin tissues were fixed in 2% paraformaldehyde for 20 s, soaked in fructose solutions, and embedded in Tissue-Tek^®^ O.C.T. Compound (Sakura Finetek USA, Inc., Torrance, CA, USA). The samples were sectioned at a thickness of 4 μm using a Leica CM1950 cryostat (Leica Biosystems, Nussloch, Germany) and mounted on glass slides for histological analysis.

### 2.5. Immunofluorescence Staining

Cryostat sections were fixed in 10% neutral-buffered formalin for 15 min at 25 °C, followed by three 5 min washes with phosphate-buffered saline (PBS). Sections were permeabilized with 0.1% Triton X-100 for 15 min and washed three times with PBS (5 min each). To block non-specific binding, sections were incubated with 1% bovine serum albumin (BSA) for 1 h at 25 °C in a humidified chamber. Primary antibodies (1:100 in 1% BSA) were applied and incubated overnight at 4 °C, followed by three PBS washes (5 min each). Sections were then incubated with fluorescent secondary antibodies (1:1000 in 1% BSA) for 1 h at 25 °C in the dark, washed three times with PBS, and mounted using DAPI-containing medium (F6057, Sigma-Aldrich). Images were captured using an OLYMPUS BX53 microscope (Olympus Corporation, Tokyo, Japan).

### 2.6. Immunoblot Assay

Back skin samples (100 mg) from each group were homogenized in 1 mL of lysis buffer containing 0.05 M Tris-HCl (pH 7.4), 1 mM EDTA, 0.15 M NaCl, 1% NP-40, 0.25% deoxycholic acid, phosphatase inhibitor cocktail 2 (P5726, Sigma-Aldrich), and protease inhibitor cocktail (S8830, Sigma-Aldrich). Homogenates were stored at −80 °C for 12 h, then centrifuged at 13,000 rpm for 1 h at 4 °C. The resulting supernatants were collected, and protein concentrations were determined using the Bradford assay (Bio-Rad Protein Assay, cat. no. 5000006, Hercules, CA, USA). Protein samples were mixed with 5× loading dye and heated at 95 °C for 5 min. Proteins were separated by SDS-PAGE (8%, 10%, or 12% gels) and transferred to polyvinylidene difluoride membranes (GE Healthcare, Amersham, UK). Membranes were blocked with 5% nonfat milk in TBST (150 mM NaCl, 20 mM Tris-HCl, pH 7.6, 0.1% Tween-20) for 1 h at 25 °C, followed by overnight incubation at 4 °C with primary antibodies. After three TBST washes, membranes were incubated with horseradish peroxidase–conjugated secondary antibodies for 1 h at 25 °C and washed again. Immunoreactive bands were visualized using Immobilon Western Chemiluminescent HRP Substrate (Millipore, Burlington, MA, USA) and imaged with an iBright Imaging System (Thermo Fisher Scientific) [26,27,28]. β-actin served as the loading control.

### 2.7. Statistical Analysis

Statistical analyses were performed using GraphPad Prism software (version 9, San Diego, CA, USA). Comparisons between two groups were conducted using Student’s *t*-test, while data from three groups were analyzed using one-way ANOVA followed by Tukey’s post hoc test. Results are presented as the mean ± standard error of the mean (SEM). Statistical significance was set at *p* < 0.05.

## 3. Results

### 3.1. Identification of Major Bioactive Compounds in the Hydroethanolic Extract of Ohwia caudata Leaves

**Figure 1:**
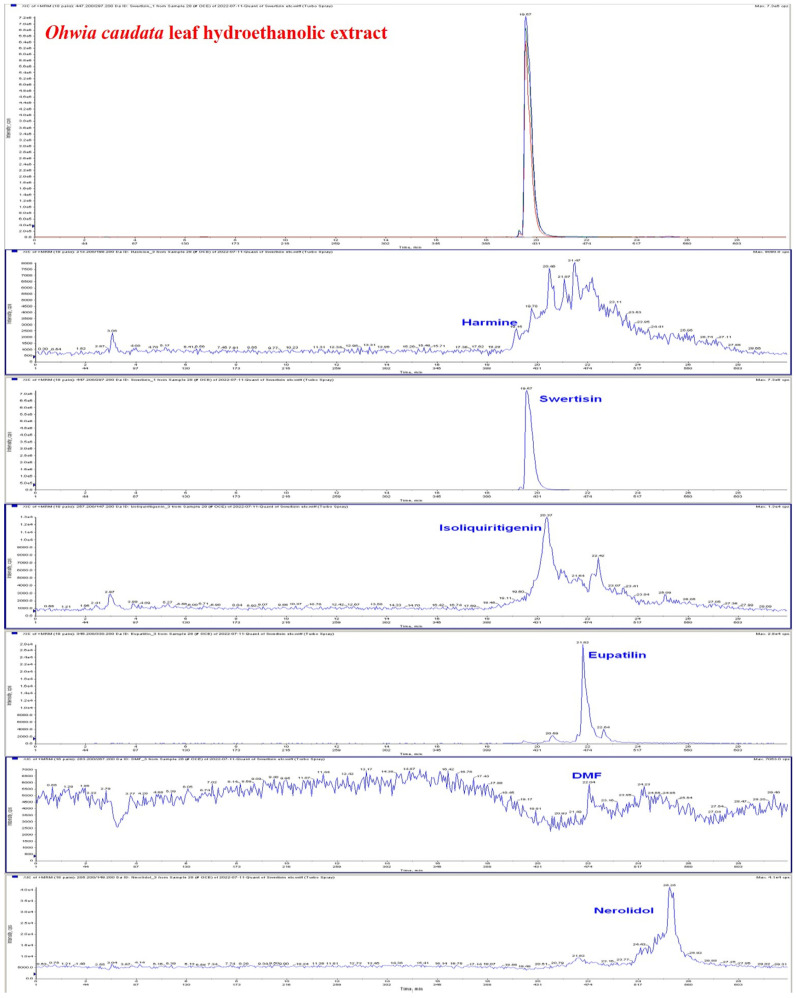
HPLC–MS chromatogram of the hydroethanolic extract of *Ohwia caudata* leaves. The extract was prepared by sonication of 10 g of dried *Ohwia caudata* leaves in 200 mL 75% ethanol for 1 h, followed by filtration and centrifugation. Analysis was performed using a high-performance liquid chromatography system coupled with tandem mass spectrometry (HPLC–MS). Major bioactive compounds identified in the extract included harmine, swertisin, isoliquiritigenin, eupatilin, 3′,4′-dimethoxyflavone (DMF), and nerolidol.

The bioactive compounds in the hydroethanolic extract of *Ohwia caudata* leaves were characterized by HPLC–MS. As shown in Figure 1, several major bioactive compounds were detected, including harmine (1.2552 ng/mL), swertisin (27,600 ng/mL), isoliquiritigenin (18.560 ng/mL), eupatilin (6.3440 ng/mL), 3′,4′-dimethoxyflavone (0.4568 ng/mL), and nerolidol (1376.0 ng/mL). The corresponding chromatographic and mass spectrometric data confirming the identification and quantification of these constituents are presented in Figure 1 and the Supplementary Material.

### 3.2. The Adverse Effects of TPA on the Skin Were Mitigated by the Ohwia caudata Leaf Hydroethanolic Extract

**Figure 2:**
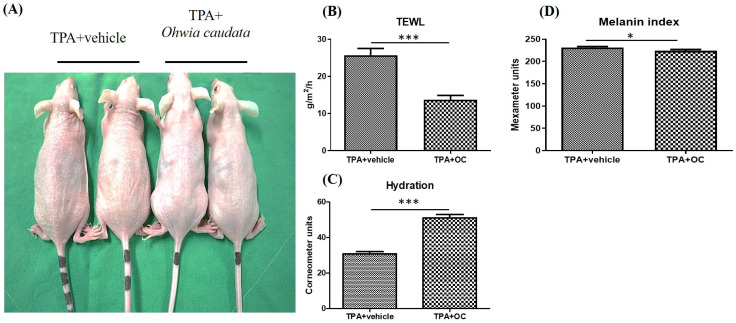
The deleterious effects of TPA on the skin were mitigated by the *Ohwia caudata* leaf hydroethanolic extract. (**A**) Representative dorsal skin images of SKH1/J hairless mice from the control, TPA-stimulated, and TPA plus extract-treated groups. (**B**–**D**) Quantitative analysis of transepidermal water loss (TEWL), stratum corneum hydration, and melanin index measured using a multi-probe adapter system (Courage + Khazaka, Cologne, Germany) after 7 days of treatment (*n* = 6 per group). Data are expressed as mean ± SEM. Statistical analysis was performed using Student’s *t*-test. * *p* < 0.05; *** *p* < 0.001. OC: *Ohwia caudata* leaf hydroethanolic extract.

Dorsal skin conditions in mice were evaluated using multiple probes (Figure 2A). TEWL, an established indicator of skin barrier integrity, was measured as the rate of water evaporation through the stratum corneum. Elevated TEWL is associated with reduced stratum corneum hydration, barrier disruption, and increased susceptibility to skin disorders such as psoriasis and atopic dermatitis [29]. TPA stimulation significantly increased TEWL and decreased stratum corneum hydration. Treatment with *Ohwia caudata* leaf hydroethanolic extract markedly attenuated these effects, resulting in reduced TEWL and improved hydration (*p* < 0.001; Figure 2B,C). In addition, TPA elevated the melanin index, whereas treatment with the extract significantly reduced melanin production (*p* < 0.05; Figure 2D). These findings indicate that *Ohwia caudata* leaf hydroethanolic extract enhances skin hydration, preserves barrier integrity, and limits melanin formation, thereby exerting a protective effect against TPA-induced acute contact dermatitis.

### 3.3. Ohwia Caudata Leaf Hydroethanolic Extract Attenuated TPA-Induced Skin Inflammation

**Figure 3:**
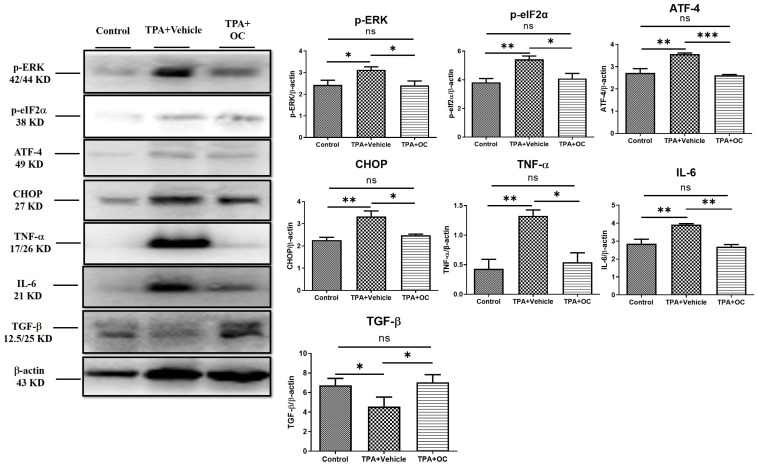
*Ohwia caudata* leaf hydroethanolic extract attenuates TPA-induced skin inflammation through suppression of the ERK signaling pathway. Dorsal skin samples from control, TPA-stimulated, and TPA plus extract-treated SKH1/J hairless mice (*n* = 3 per group) were analyzed by immunoblotting. Protein expression levels of IL-6, TNF-α, TGF-β, phosphorylated ERK (p-ERK), phosphorylated eIF2α (p-eIF2α), ATF-4, and CHOP were evaluated, with β-actin used as a loading control. Data represent mean ± SEM from three independent experiments. Statistical analysis was performed using one-way ANOVA followed by Tukey’s multiple comparisons test. ns: no significant difference; * *p* < 0.05; ** *p* < 0.01; *** *p* < 0.001. OC: *Ohwia caudata* leaf hydroethanolic extract.

**Figure 4:**
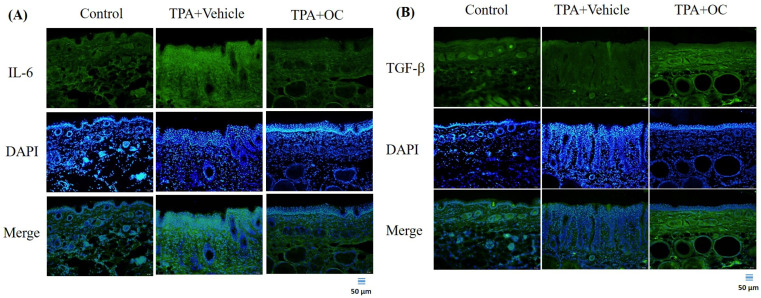
The *Ohwia caudata* leaf hydroethanolic extract modulates IL-6 and TGF-β expression in TPA-induced dermatitis. Dorsal skin cryosections (4 μm thick) from control, TPA-stimulated, and TPA plus extract-treated SKH1/J hairless mice were analyzed by immunofluorescence staining. (**A**) IL-6 and (**B**) TGF-β signals were visualized using Alexa Fluor^®^ 488-conjugated secondary antibodies, and nuclei were counterstained with DAPI. Images were captured under an OLYMPUS BX53 fluorescence microscope. Scale bar = 50 μm. OC: *Ohwia caudata* leaf hydroethanolic extract. The target proteins were visualized using green fluorescence and the cell nuclei were stained with blue fluorescence (DAPI).

Immunoblotting and immunofluorescence staining were performed to assess inflammatory cytokines in dorsal skin, which showed no significant changes in the protein microarray. TPA stimulation markedly increased IL-6 (*p* < 0.01) and TNF-α (*p* < 0.01) expression compared with the control group, whereas treatment with *Ohwia caudata* leaf hydroethanolic extract reversed these elevations (IL-6, *p* < 0.01; TNF-α, *p* < 0.05 compared with the TPA-stimulated group; Figure 3). Immunofluorescence images further confirmed that IL-6 signals were elevated in TPA-treated skin and reduced following extract administration (Figure 4A). These results demonstrate that *Ohwia caudata* leaf hydroethanolic extract suppresses TPA-induced proinflammatory cytokine expression, thereby mitigating acute contact dermatitis. In addition, TGF-β, a key immunoregulatory cytokine involved in repressing immune responses [30,31], was examined. Both immunoblotting and immunofluorescence analyses also showed that TPA stimulation reduced TGF-β expression (*p* < 0.05) compared with the control group, whereas treatment with the extract restored TGF-β levels (*p* < 0.05 compared with the TPA-stimulated group; Figure 3 and Figure 4B). Restoration of this cytokine suggests that the extract modulates immune balance in addition to suppressing proinflammatory cytokines. Collectively, these findings indicate that *Ohwia caudata* leaf hydroethanolic extract exerts anti-inflammatory effects through a dual mechanism: downregulation of IL-6 and TNF-α and restoration of TGF-β expression, thereby alleviating TPA-induced skin inflammation.

### 3.4. The ERK Signaling Pathway Was Attenuated by Ohwia caudata Leaf Hydroethanolic Extract

**Figure 5:**
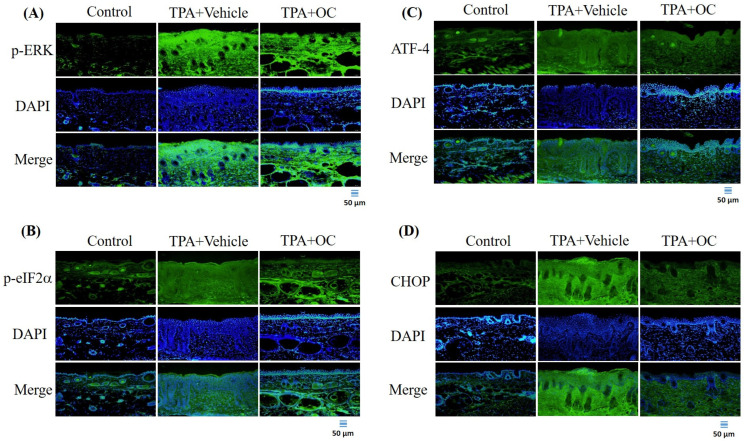
The *Ohwia caudata* leaf hydroethanolic extract suppresses ERK pathway activation in TPA-treated skin. Immunofluorescence staining of dorsal skin sections (4 μm thick) from control, TPA-stimulated, and TPA plus extract-treated SKH1/J hairless mice was performed to detect phosphorylated ERK (**A**), phosphorylated eIF2α (**B**), ATF-4 (**C**), and CHOP (**D**). Alexa Fluor^®^ 488-conjugated antibodies were used for detection, and nuclei were counterstained with DAPI. Images were acquired using an OLYMPUS BX53 fluorescence microscope. Scale bar = 50 μm. OC: *Ohwia caudata* leaf hydroethanolic extract. The target proteins were visualized using green fluorescence and the cell nuclei were stained with blue fluorescence (DAPI).

Previous studies have indicated that the ERK signaling pathway contributes to the pathogenesis of atopic dermatitis and that suppression of ERK activity alleviates disease severity [32]. Furthermore, ERK activation can be triggered by TPA stimulation [33,34]. To investigate this pathway, the p-ERK level was analyzed by immunoblotting and immunofluorescence staining. TPA treatment significantly increased p-ERK expression (*p* < 0.05) compared with the control group, whereas this elevation was attenuated following *Ohwia caudata* leaf hydroethanolic extract treatment (*p* < 0.05 compared with the TPA-stimulated group; Figure 3 and Figure 5A). The expression of downstream ERK-regulated proteins—including p-eIF-2α [35], ATF-4 [35,36], and CHOP [36]—was also examined. TPA stimulation markedly increased all three proteins (p-eIF-2α, *p* < 0.01; ATF-4, *p* < 0.01; CHOP, *p* < 0.01) compared with the control group, whereas treatment with *Ohwia caudata* leaf hydroethanolic extract significantly reduced their expression (p-eIF-2α, *p* < 0.05; ATF-4, *p* < 0.001; CHOP, *p* < 0.05 compared with the TPA-stimulated group; Figure 3 and Figure 5B–D). These results indicate that *Ohwia caudata* leaf hydroethanolic extract reduces TPA-induced activation of the ERK signaling pathway in the skin.

### 3.5. Ohwia caudata Leaf Hydroethanolic Extract Enhanced SIRT1/Nrf2/HO-1 Antioxidant Signaling in TPA-Stimulated Skin

**Figure 6:**
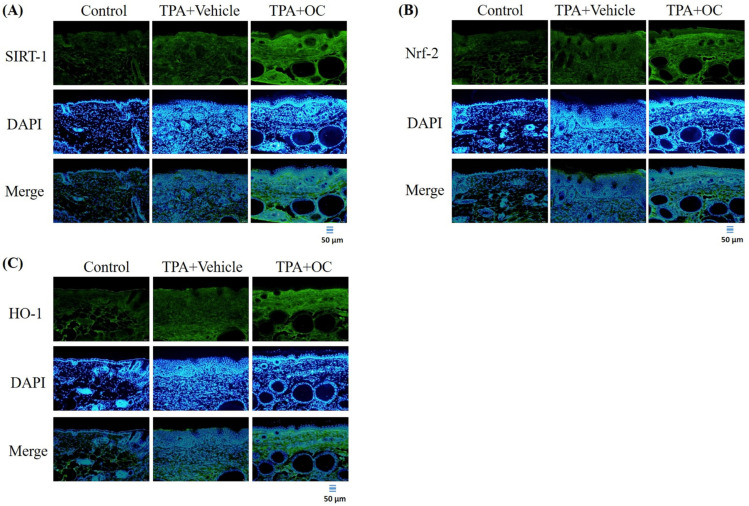
*Ohwia caudata* leaf hydroethanolic extract activates the SIRT1/Nrf2/HO-1 antioxidative pathway in TPA-induced dermatitis. Immunofluorescence staining of dorsal skin sections (4 μm thick) from control, TPA-stimulated, and TPA plus extract-treated SKH1/J hairless mice (*n* = 6 per group) was conducted to assess (**A**) SIRT1, (**B**) Nrf2, and (**C**) HO-1 expression. Alexa Fluor^®^ 488-conjugated antibodies were used, and nuclei were counterstained with DAPI. Fluorescence images were captured using an OLYMPUS BX53 microscope. Scale bar = 50 μm. OC: *Ohwia caudata* leaf hydroethanolic extract. The target proteins were visualized using green fluorescence and the cell nuclei were stained with blue fluorescence (DAPI).

**Figure 7:**
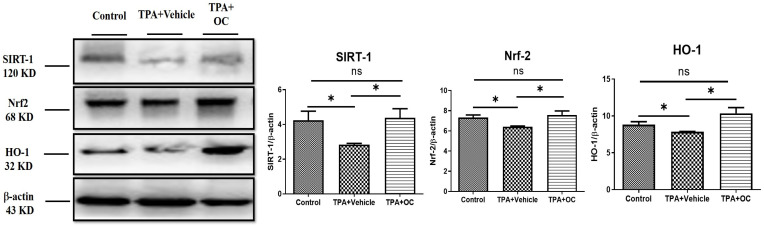
*Ohwia caudata* leaf hydroethanolic extract enhances antioxidant protein expression in TPA-induced dermatitis. Dorsal skin lysates from control, TPA-stimulated, and TPA plus extract-treated SKH1/J hairless mice (*n* = 3 per group) were analyzed by immunoblotting for SIRT1, Nrf2, and HO-1 expression. β-actin served as a loading control. Densitometric values are expressed as mean ± SEM from three independent experiments. Statistical analysis was performed using one-way ANOVA followed by Tukey’s multiple comparisons test. ns: not significant; * *p* < 0.05. OC: *Ohwia caudata* leaf hydroethanolic extract.

Oxidative stress plays a critical role in the onset and progression of various inflammatory skin conditions. Consequently, strategies that minimize oxidative damage and restore redox balance offer a promising therapeutic approach to managing these conditions [37]. Given that SIRT1 mediates Nrf2-dependent antioxidant signaling [28], the expression of SIRT1, Nrf2, and their downstream effector HO-1 was examined by immunofluorescence staining and immunoblot assay. As shown in Figure 6, treatment with *Ohwia caudata* leaf hydroethanolic extract increased SIRT1 and Nrf2 fluorescence signals in the epidermis, accompanied by enhanced HO-1 expression. Consistent with these findings, immunoblot analysis (Figure 7) revealed elevated protein levels of SIRT1, Nrf2, and HO-1 in the extract-treated group compared with the TPA-stimulated group. Quantitative analysis confirmed significant increases in these proteins following extract treatment (SIRT1, *p* < 0.05; Nrf2, *p* < 0.05; HO-1, *p* < 0.05). These results suggest that the extract enhances antioxidant signaling through activation of the SIRT1/Nrf2/HO-1 pathway.

## 4. Discussion

This study elucidates the protective and therapeutic potential of *Ohwia caudata* leaf hydroethanolic extract against TPA-induced acute contact dermatitis, demonstrating its ability to act through multiple mechanisms to restore skin health. The skin barrier constitutes the primary defense system of humans, providing both a physical and biochemical shield that prevents the invasion of pathogens, allergens, and environmental toxins. A critical determinant of barrier integrity is TEWL, which has been widely established as a reliable biomarker for assessing skin barrier function, particularly under conditions of injury, inflammation, or disease. Increased TEWL reflects a compromised epidermal barrier, typically characterized by impaired stratum corneum function, heightened permeability, and reduced resistance to external irritants—all of which contribute to the progressive deterioration of skin health. Conversely, reduced TEWL is generally indicative of barrier recovery, improved epidermal cohesion, and restored physiological balance within the skin [38]. In addition to TEWL, skin hydration plays a pivotal role in maintaining epidermal flexibility, enzymatic activity, and stratum corneum integrity. Adequate water retention is indispensable for sustaining barrier homeostasis [39]. In this study, treatment with *Ohwia caudata* leaf hydroethanolic extract markedly attenuated the deleterious effects of TPA exposure, as evidenced by a significant reduction in TEWL accompanied by restoration of stratum corneum hydration. These findings suggest that *Ohwia caudata* not only limits barrier disruption but also actively promotes barrier repair and rehydration, thereby reinforcing epidermal resilience against external insults. Beyond its barrier-protective effects, *Ohwia caudata* extract also suppressed melanin production, further underscoring its multifaceted role in maintaining skin homeostasis. Excessive melanin synthesis is often associated with oxidative stress and inflammatory signaling, both of which are exacerbated under pathological conditions such as dermatitis [40]. Thus, the ability of *Ohwia caudata* to modulate melanogenesis may provide an additional protective mechanism that prevents hyperpigmentation and promotes more uniform skin recovery following inflammatory damage. These results indicate that *Ohwia caudata* leaf hydroethanolic extract exerts comprehensive protective effects on the skin barrier by reducing TEWL, enhancing hydration, and modulating pigmentation.

The present study also demonstrated the anti-inflammatory properties of *Ohwia caudata* leaf hydroethanolic extract in a TPA-induced dermatitis model. TPA stimulation significantly increased the expression of pro-inflammatory cytokines, including TNF-α and IL-6, which are well-established mediators of skin inflammation. Previous studies have shown that chronic TPA exposure persistently elevates TNF-α and IL-6 levels, thereby exacerbating cutaneous inflammation and tissue damage [41]. Reducing the expression of these cytokines is therefore critical for alleviating inflammatory responses. In this study, treatment with *Ohwia caudata* leaf hydroethanolic extract markedly attenuated the TPA-induced elevation of TNF-α and IL-6. In addition, the extract restored levels of TGF-β, a key immunoregulatory cytokine involved in maintaining immune tolerance and tissue homeostasis [30]. These findings suggest that *Ohwia caudata* exerts a dual immunomodulatory effect by suppressing pro-inflammatory mediators while restoring anti-inflammatory regulation, thereby conferring a comprehensive anti-inflammatory benefit.

The ERK signaling pathway was also found to be involved in the protective effects of *Ohwia caudata*. Previous research has established the pivotal role of ERK signaling in the pathogenesis of atopic dermatitis, with suppression of ERK activity being associated with reduced disease severity [32]. Importantly, TPA has been reported to activate ERK signaling, further linking this pathway to inflammation-associated skin pathology [33,34]. Moreover, ERK activation can trigger downstream signaling cascades, including the eIF-2α/ATF-4/CHOP axis, which amplifies the release of inflammatory mediators and contributes to tissue injury [35,42,43]. In this study, TPA stimulation led to elevated levels of phosphorylated ERK and its downstream targets (eIF-2α, ATF-4, and CHOP). Notably, treatment with *Ohwia caudata* leaf hydroethanolic extract significantly reduced the phosphorylation of these proteins, indicating effective attenuation of ERK pathway activation. This suppression of ERK signaling and its downstream pro-inflammatory effectors highlight a potential therapeutic mechanism by which *Ohwia caudata* reduces skin inflammation and mitigates contact dermatitis.

In addition to inflammation, oxidative stress plays a fundamental role in the pathogenesis of inflammatory skin diseases. Excessive production of reactive oxygen species disrupts the cellular redox balance, aggravates inflammatory signaling, and accelerates tissue damage [37,44]. Thus, targeting oxidative stress represents an important therapeutic strategy for managing skin inflammation. Among the critical regulators of oxidative stress, SIRT1 has been identified as a key upstream activator of the Nrf2 antioxidative signaling pathway. Activation of Nrf2 enhances the transcription of antioxidant defense genes, including HO-1, thereby strengthening the cellular capacity to neutralize reactive oxygen species and restore redox homeostasis [28]. In this study, *Ohwia caudata* leaf hydroethanolic extract significantly upregulated the expression of SIRT1, Nrf2, and HO-1, underscoring its antioxidative potential. By enhancing this axis, the extract not only counteracts oxidative stress but also indirectly suppresses inflammation, given the close interplay between oxidative imbalance and inflammatory signaling.

These findings indicate that *Ohwia caudata* leaf hydroethanolic extract exerts multifaceted therapeutic effects against TPA-induced dermatitis. Its actions include suppression of pro-inflammatory cytokines, restoration of TGF-β, inhibition of ERK and its downstream signaling, and activation of the SIRT1/Nrf2/HO-1 antioxidative pathway. These combined effects suggest that *Ohwia caudata* can both reduce inflammatory responses and enhance cellular defenses against oxidative stress, thereby providing a strong rationale for its development as a potential therapeutic agent for contact dermatitis and other inflammatory skin disorders.

The HPLC–MS analysis revealed that the hydroethanolic extract of *Ohwia caudata* leaves contains several bioactive constituents, including harmine, swertisin, isoliquiritigenin, eupatilin, 3′,4′-dimethoxyflavone, and nerolidol. Most of these compounds have been reported to possess potent antioxidant and anti-inflammatory properties, which may collectively contribute to the observed biological activities of the extract. Specifically, harmine, swertisin, isoliquiritigenin, eupatilin, and nerolidol have all been documented to attenuate oxidative stress and inflammatory responses through diverse molecular mechanisms [45,46,47,48,49,50,51]. Moreover, harmine and isoliquiritigenin are known to regulate melanin synthesis, suggesting potential applications in pigmentation modulation and skin-brightening formulations [52,53]. In addition, eupatilin has demonstrated efficacy in alleviating psoriasis-like skin lesions by modulating inflammatory signaling pathways [54]. Notably, nerolidol has been identified as a skin penetration enhancer, facilitating the transdermal absorption of active compounds and improving bioavailability [51]. The presence of these multifunctional phytochemicals in *Ohwia caudata* leaf hydroethanolic extract underscores its potential as a natural source of antioxidant, anti-inflammatory, and dermatoprotective agents, supporting its possible use in skincare and the management of dermatitis.

Additionally, although murine models are widely used to investigate human skin diseases, the protective and therapeutic effects of *Ohwia caudata* leaf hydroethanolic extract observed in this study should be interpreted with caution, given the structural and physiological differences between murine and human skin. These differences include variations in epidermal architecture, hydration, follicular density, mechanical elasticity, subcutaneous structure, immune regulation, and regenerative processes—all of which may influence the biological response to topical treatment [55,56,57,58]. Consequently, further research using human-relevant models, such as ex vivo human skin assays or controlled clinical trials, is required to rigorously validate the efficacy, safety, and dermal bioavailability of the extract. Such studies are essential to strengthen the translational potential of these findings and support the rational development of *Ohwia caudata* leaf hydroethanolic extract as a novel therapeutic for human dermatological conditions.

Despite the promising results, several limitations should be acknowledged. First, the experiments were conducted in an acute murine model of TPA-induced contact dermatitis, which may not fully capture the complexity of human skin physiology or chronic dermatological conditions. Further validation using human skin models or clinical investigations is warranted. Second, the extract used was a crude hydroethanolic preparation containing multiple phytochemicals, and the specific active constituents responsible for the observed anti-inflammatory and antioxidant effects remain unidentified. Third, only a single concentration, small sample size (*n* = 6 per group), and short-term exposure were evaluated, which may limit statistical power and interpretation of dose dependence, long-term efficacy, and safety. Fourth, while all animals were randomly assigned and maintained under controlled conditions, detailed data on randomization procedures, data distribution, and potential confounding factors such as batch variability or environmental influences were not fully explored. Fifth, mechanistic analyses were also limited to the ERK and SIRT1/Nrf2/HO-1 signaling pathways without investigating upstream regulators or cross-talk with other pathways such as NF-κB or MAPK. Sixth, pharmacokinetic profiling and topical absorption efficiency were not assessed, which may affect translational relevance. Finally, potential variations in extract composition due to environmental or batch-related factors were not assessed. Addressing these limitations in future studies will strengthen mechanistic understanding and support the translational development of *Ohwia caudata*–based therapeutics.

In conclusion, this study provides substantial evidence that *Ohwia caudata* leaf hydroethanolic extract exerts multifaceted protective effects against TPA-induced skin damage. By restoring skin barrier integrity, reducing inflammation, modulating immune responses, and enhancing antioxidative capacity, the extract demonstrates significant potential as a therapeutic agent for inflammatory skin conditions such as acute contact dermatitis.

## 5. Conclusions

The present study provides compelling evidence that the hydroethanolic extract of *Ohwia caudata* leaves exerted therapeutic effects against TPA-induced acute contact dermatitis in murine models. Treatment with the extract markedly improved skin barrier integrity, as indicated by reduced TEWL and enhanced stratum corneum hydration. In addition, the extract demonstrated potent anti-inflammatory activity by downregulating pro-inflammatory cytokines (IL-6, TNF-α) and restoring the levels of the regulatory cytokine TGF-β. Mechanistically, these effects were mediated through the attenuation of the ERK signaling pathway and its downstream targets, including p-eIF-2α, ATF-4, and CHOP. Furthermore, the extract activated the SIRT1/Nrf2/HO-1 axis, underscoring its antioxidative potential in mitigating oxidative stress associated with inflammatory skin damage. Collectively, these findings suggest that the hydroethanolic extract of *Ohwia caudata* leaves represents a promising natural therapeutic agent for the prevention and treatment of inflammatory skin conditions such as acute contact dermatitis. Future research should focus on elucidating its molecular targets and evaluating its efficacy in clinical settings.

## Data Availability

The authors confirmed that the data supporting the findings of this study are available within the article. The raw data used and/or analyzed during the current study are available from the corresponding author on request.

## Supplementary Materials

The following supporting information can be downloaded at: https://www.mdpi.com/article/10.3390/life15111707/s1, HPLC–MS Original Report.

## Abbreviations

The following abbreviations are used in this manuscript:

ATF-4	activating transcription factor 4
CHOP	C/EBP homologous protein
p-eIF2α	phosphorylated eukaryotic initiation factor 2
p-ERK	phosphorylated extracellular signal-regulated kinase
HO-1	heme oxygenase-1
HPLC–MS	high-performance liquid chromatography coupled with tandem mass spectrometry
IL-6	interleukin-6
Nrf2	nuclear factor erythroid 2–related factor 2
SIRT1	Sirtuin 1
TEWL	Transepidermal water loss
TGF-β	transforming growth factor beta
TNF-α	tumor necrosis factor-alpha
TPA	12-O-tetradecanoylphorbol-13-acetate
