# Correction: Lin et al. Protective Anti-Inflammatory and Antioxidant Mechanisms of *Ohwia caudata* Leaf Hydroethanolic Extract in a Dermatitis Mouse Model. *Life* 2025, *15*, 1707

**DOI:** 10.3390/life16091416

**Published:** 2026-08-26

**Authors:** Tzu-Kai Lin, Bruce Chi-Kang Tsai, Shih-Wen Kao, Chia-Lun Tsai, Chia-Hua Kuo, Tsung-Jung Ho, Dennis Jine-Yuan Hsieh, Shinn-Zong Lin, Wei-Wen Kuo, Chih-Yang Huang

**Affiliations:** 1 Department of Dermatology, Hualien Tzu Chi Hospital, Buddhist Tzu Chi Medical Foundation, Hualien 970473, Taiwan; 2 Department of Dermatology, School of Medicine, Tzu Chi University, Hualien 970374, Taiwan; 3 Cardiovascular and Mitochondrial Related Disease Research Center, Hualien Tzu Chi Hospital, Buddhist Tzu Chi Medical Foundation, Hualien 970473, Taiwan; 4 Department of Orthopedic Surgery, Chung Shan Medical University Hospital, Taichung 402306, Taiwan; 5 School of Medicine, Chung Shan Medical University, Taichung 402306, Taiwan; 6 Laboratory of Exercise Biochemistry, The Education University of Hong Kong, New Territories, Hong Kong; 7 School of Physical Education and Sports Science, Soochow University, Suzhou 215006, China; 8 Department of Movement Sciences and Sports Training, School of Sport Sciences, University of Jordan, Amman 11942, Jordan; 9 Department of Chinese Medicine, Hualien Tzu Chi Hospital, Buddhist Tzu Chi Medical Foundation, Hualien 970473, Taiwan; 10 Integration Center of Traditional Chinese and Modern Medicine, Hualien Tzu Chi Hospital, Buddhist Tzu Chi Medical Foundation, Hualien 970473, Taiwan; 11 Department of Medical Laboratory and Biotechnology, Chung Shan Medical University, Taichung 402306, Taiwan; 12 Clinical Laboratory, Chung Shan Medical University Hospital, Taichung 402306, Taiwan; 13 Bioinnovation Center, Buddhist Tzu Chi Medical Foundation, Hualien 970473, Taiwan; 14 Department of Neurosurgery, Hualien Tzu Chi Hospital, Buddhist Tzu Chi Medical Foundation, Hualien 970473, Taiwan; 15 Department of Biological Science and Technology, College of Life Sciences, China Medical University, Taichung 406040, Taiwan; 16 Ph.D. Program for Biotechnology Industry, China Medical University, Taichung 406040, Taiwan; 17 School of Pharmacy, China Medical University, Taichung 406040, Taiwan; 18 Graduate Institute of Biomedical Sciences, China Medical University, Taichung 404333, Taiwan; 19 Department of Medical Research, China Medical University Hospital, China Medical University, Taichung 404327, Taiwan; 20 Department of Medical Laboratory Science and Biotechnology, Asia University, Taichung 413305, Taiwan

In the published publication [1], there was an error regarding the affiliation for “Tzu-Kai Lin”. In addition to “affiliation no. 1”, the updated affiliation should include: “Department of Dermatology, School of Medicine, Tzu Chi University, Hualien 970374, Taiwan”. The authors state that the scientific conclusions are unaffected. This correction was approved by the Academic Editor. The original publication has also been updated.
