# Supplementary material for: Protective Anti-Inflammatory and Antioxidant Mechanisms of Ohwia caudata Leaf Hydroethanolic Extract in a Dermatitis Mouse Model
Source: Life (Basel). 2025 Nov 4;15(11):1707. doi: 10.3390/life15111707 (PMC12653036; doi:10.3390/life15111707)
Supplement: Supplementary file 1 [file life-15-01707-s001.zip › life-3906596-supplementary.pdf]

**Supplementary Material**

**HPLC-MS results:**

***Ohwia caudata*** **leaf hydroethanolic extract**

**Instrument Device :**

| Instrument Categories | Instrument Model |
|---|---|
| Pump Model | Agilent 1260 G1312B Binary Pump |
| AutoSampler | Agilent 1260 G1367E HiP ALS Autosampler |
| Degasser | Agilent 1260 G1379B Degasser |
| Column | Phenomenex Kinetex-Phenyl-Hexyl-100A (100 mm x 2.1 mm i.d., 2.6 um) |
| Mass Spectrometer | AB Sciex Instruments QTRAP 5500 |
| Source type | Turbo V Ion Source |

**HPLC Method Properties :**

Duration : 30 min
Injection volume : 5 ul
Mobile Phase : A: 0.1% ( v/v ) Formic acid / Water ; B: 0.1% ( v/v ) Formic acid / Acetonitrile
Step Table :

| Step | Total Time(min) | Flow Rate(μl/min) | A (%) | B (%) |
|---|---|---|---|---|
| 0 | 0.10 | 100 | 95.0 | 5.00 |
| 1 | 5.00 | 100 | 95.0 | 5.00 |
| 2 | 8.00 | 100 | 50.0 | 50.0 |
| 3 | 10.0 | 100 | 5.00 | 95.0 |
| 4 | 15.0 | 100 | 5.00 | 95.0 |
| 5 | 16.0 | 100 | 95.0 | 5.00 |
| 6 | 25.0 | 100 | 95.0 | 5.00 |

**Sample Preparation :**

A volume of 30 µL of the sample was taken, followed by the addition of 90 µL of methanol. The mixture was vortexed thoroughly to ensure complete dissolution and subsequently incubated at -20°C for 30 minutes. Centrifugation was performed at 15,000 × g for 10 minutes, and the supernatant was collected and diluted 2× with 50% methanol prior to analysis. (Total dilution factor: 8×)

**Mass Spectrometer Information :**

Scan Type : MRM (Multiple Reaction Monitor)
Polarity : Positive ion mode
Source temperature : 500 ℃
Data acquisition : Analyst 1.5 software

**Parameter Table :**

CUR (curtain gas) : 20.00 psi Nebulizing gas ( GS1 ) : 40.00 psi
Collision-activated Dissociation (CAD) : High Heating gas ( GS2 ) : 50.00 psi
Electrospray capillary voltage : 5000.00 V

**Preparation of Calibrators :**
The powdered standard was dissolved in 50% methanol to prepare stock solutions of each target compound. These stock solutions were then used to prepare mixed working solutions with concentrations around μg/mL, which were stored at -20°C for future use. Calibration solutions were freshly prepared and serially diluted with 50% methanol to achieve a concentration range of μg/mL ~ ng/mL.

**Quantitation Method :**
As the provided standards are not isotope-labeled, the Multiple Point External Standard method was employed for quantitation.

**Selected Transitions and Parameter Settings**

| Analytes | Q1 mass | Q3 mass | DP (V) | EP (V) | CE (V) | CXP (V) |
|---|---|---|---|---|---|---|
| Harmine | 213.2 | 144.2<br>170.2<br>198.2 | 220 | 10 | 56<br>43<br>31 | 16<br>13<br>19 |
| Swertisin | 447.2 | 297.2<br>381.2<br>429.2 | 90 | 10 | 34<br>22<br>18 | 50<br>26<br>35 |
| Isoliquiritigenin | 257.2 | 119.2<br>137.2<br>147.2 | 25 | 10 | 38<br>35<br>26 | 13<br>10<br>13 |
| Eupatilin | 345.2 | 169.2<br>315.3<br>330.2 | 120 | 10 | 50<br>43<br>34 | 13<br>22<br>28 |
| 3',4'-Dimethoxyflavone (DMF) | 283.2 | 222.2<br>239.2<br>267.2 | 130 | 10 | 34<br>37<br>35 | 20<br>22<br>22 |
| Nerolidol | 205.2 | 109.2<br>121.2<br>149.2 | 30 | 10 | 20<br>19<br>14 | 15<br>22<br>11 |

**Note:** Red indicates the ion pairs used for quantitation.

## Calibration Curve of Standard

**Linear regression** **Weighting：1/x**

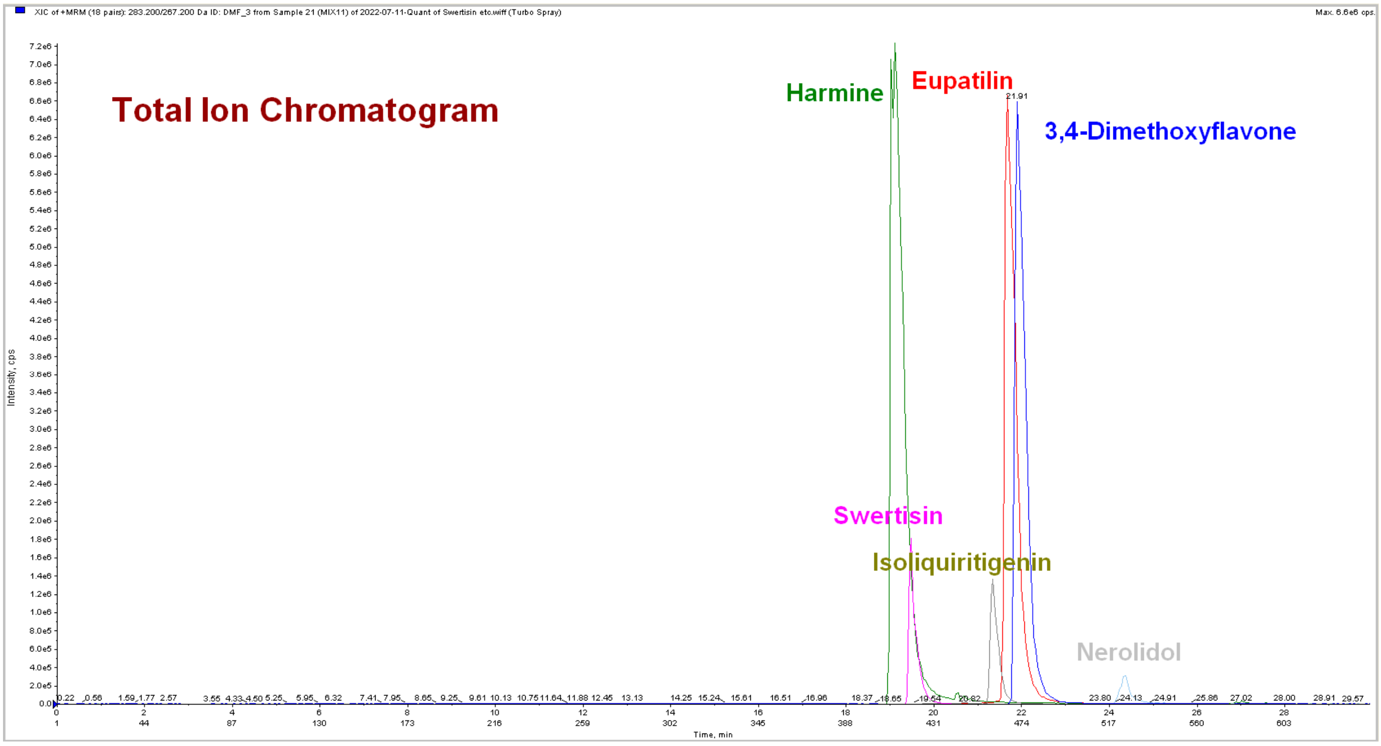


Note:

1. The delayed precipitation of Harmine and Swertisin is likely due to column aging but does not affect quantification.
2. The nerolidol signal shows low sensitivity and is easily masked by stronger signals.
3. Selected ion pairs yield distinct signals at the same retention time, confirming specificity.
4. Because of sample complexity and variable compound levels, individual XIC plots are shown rather than a single chromatogram.

## Sample Spectrum

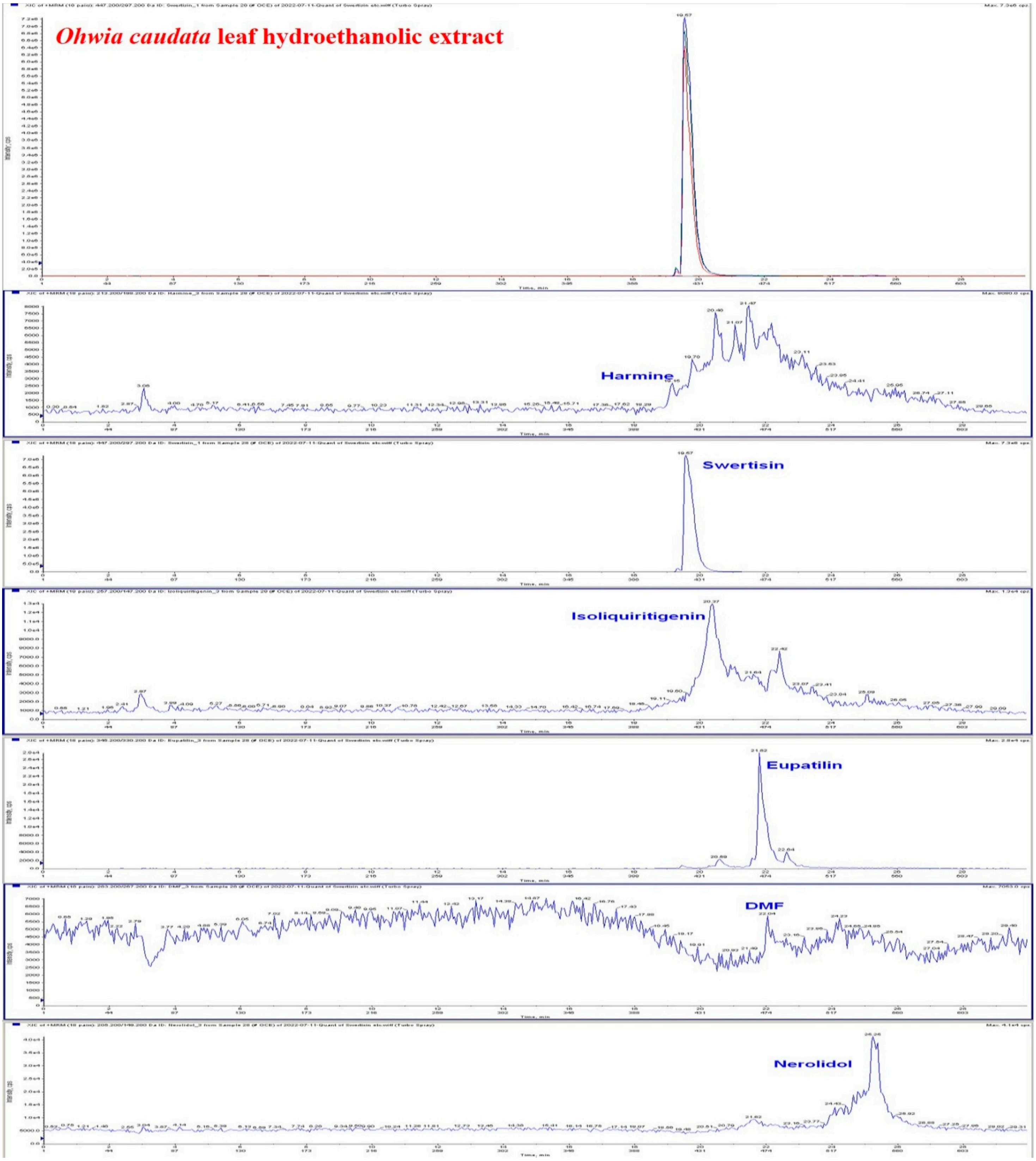

## Results (unit: ng/mL)

| Analyte Name | Spectrum | Calculated concentration ( ng/mL ) |
|---|---|---|
| Harmine | 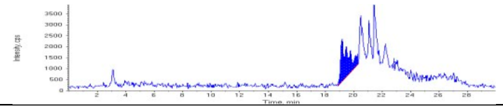 | 0.1569 (25.98%) |
| Swertisin | 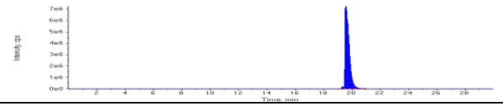 | 3450.0 |
| Isoliquiritigenin | 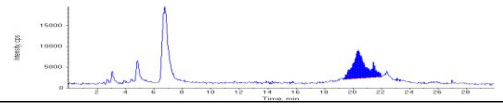 | 2.3200 |
| Eupatilin | 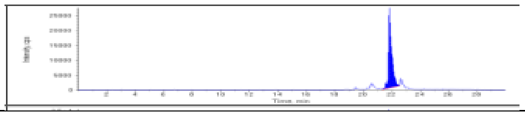 | 0.7930 |
| 3’,4’-Dimethoxyflavone (DMF) | 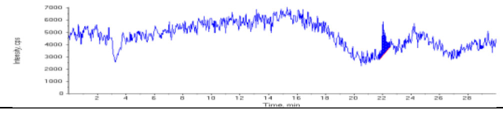 | 0.0571 (36.61%) |
| Nerolidol | 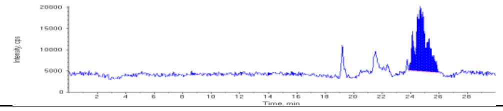 | 172.00 |

The original analysis report contained results for multiple extracts, but the present report only summarizes the data of *Ohwia caudata* leaves hydroethanolic extract. Although signals for harmine and 3′,4′-dimethoxyflavone were detected in *Ohwia caudata* leaves hydroethanolic extract, their integrated peak areas were below the lower limit of quantification, and thus the software could not convert them into quantifiable values. For reference, the baseline values from the other extracts (harmine: 0.6040 ng/mL [100%]; 3′,4′-dimethoxyflavone: 0.1560 ng/mL [100%]) are used here to calculate the concentration of these compounds for the *Ohwia caudata* leaves hydroethanolic extract.

**Summary (The precise concentration of each compound in the sample; unit : ng/mL)**

**Calculation Formula:** Analyte calculated concentration × 8 (dilution factor)

| Analyte Name | Sample |
|---|---|
| Harmine | 1.2552 |
| Swertisin | 27600 |
| Isoliquiritigenin | 18.560 |
| Eupatilin | 6.3440 |
| 3’,4’-Dimethoxyflavone (DMF) | 0.4568 |
| Nerolidol | 1376.0 |
